# Basal Ganglia Atrophy and Impaired Cognitive Processing Speed in Multiple Sclerosis

**DOI:** 10.7759/cureus.52603

**Published:** 2024-01-20

**Authors:** Tetsuya Akaishi, Juichi Fujimori, Ichiro Nakashima

**Affiliations:** 1 Department of Education and Support for Regional Medicine, Tohoku University Hospital, Sendai, JPN; 2 Department of Neurology, Tohoku Medical and Pharmaceutical University, Sendai, JPN

**Keywords:** putamen, multiple sclerosis, cognitive processing speed, brain atrophy, basal ganglia

## Abstract

Impaired cognitive processing speed is among the important higher brain dysfunctions in multiple sclerosis (MS). However, the exact structural mechanisms of the dysfunction remain uncertain. This study aimed to identify the brain regions associated with the impaired cognitive processing speed in MS by comparing the cognitive processing speed, measured using the Cognitive Processing Speed Test (CogEval) z-score, and brain regional volumetric data. Altogether, 80 patients with MS (64 with relapsing-remitting MS [RRMS] and 16 with secondary progressive MS [SPMS]) were enrolled. Consequently, CogEval z-scores were worse in patients with SPMS than in those with RRMS (p=0.001). In the univariate correlation analyses, significant correlations with CogEval z-score were suggested in the MS lesion volume (p<0.001; Spearman’s rank correlation test) and atrophies in the cerebral cortex (p=0.031), cerebral white matter (p=0.013), corpus callosum (p=0.001), thalamus (p=0.001), and putamen (p<0.001). Multiple linear regression analysis revealed that putamen atrophy was significantly associated with CogEval z-score (p=0.038) independent of volume in other brain regions, while thalamic atrophy was not (p=0.79). Univariate correlation analyses were further performed in each of RRMS and SPMS. None of the evaluated volumetric data indicated a significant correlation with the CogEval z-score in RRMS. Meanwhile, atrophies in the cerebral white matter (p=0.008), corpus callosum (p=0.002), putamen (p=0.011), and pallidum (p=0.017) demonstrated significant correlations with CogEval z-score in SPMS. In summary, the putamen could be an important region of atrophy contributing to the impaired cognitive speed in MS, especially in the later disease stages after a transition to SPMS.

## Introduction

Cognitive impairment (CI) is among the common neurological manifestations of multiple sclerosis (MS). Overall, 40%-70% of patients with MS are thought to experience neuropsychological impairments in one or more cognitive domains [1]. This could cause disturbances in a wide variety of cognitive functions, such as information-processing speed (IPS), working and episodic memory, executive functions, visuospatial abilities, and attention; however, language is rarely involved in MS [2]. IPS in MS is commonly evaluated with cognitive tests, such as the Symbol Digit Modalities Test (SDMT), Paced Auditory Serial Addition Test (PASAT), or Processing Speed Test (PST). In routine clinical care for patients with MS, SDMT or a similarly validated test has been recommended as a screening tool [3]. To date, evidence of the involvement of deep gray matter structures in the impaired IPS among patients with MS is increasing. In previous studies, atrophy in the thalamus was found to be linked with poor performance in IPS tasks such as SDMT and PASAT [1]. However, most previous studies have focused on the neural substrate for the results of cognitive tests in early disease stages. The exact brain regions of atrophy primarily contributing to impaired cognitive processing speed in the long-term progression of MS remain uncertain. Therefore, the present study aimed to elucidate the brain regions playing primary roles in the development of IPS impairment over a longer period of time by evaluating data from patients in both disease stages of relapsing-remitting MS (RRMS) and secondary progressive MS (SPMS).

## Materials and methods

Participants

The present study recruited patients with MS who visited the Department of Neurology at Tohoku Medical and Pharmaceutical University Hospital, Sendai, Japan, in 2021 and were evaluated using both the Cognitive Processing Speed Test (CogEval) and brain volumetric measurements. Individuals who fulfilled the following criteria were initially recruited: (1) diagnosed with MS based on the 2017 revision of the McDonald criteria [4] and (2) aged between 20 and 70 years. Individuals with the following backgrounds were excluded: positivity for anti-aquaporin 4 antibody and/or anti-myelin oligodendrocyte glycoprotein antibody in serum and/or cerebrospinal fluid with a cell-based assay and a history of psychiatric illness other than stable depressive symptoms [5]. Those who were diagnosed with primary progressive MS (PPMS) at the volumetric evaluations were further excluded.

CogEval z-scores

IPS was evaluated using an iPad-based screening PST, CogEval (Biogen Inc., Cambridge, MA) (https://apps.apple.com/us/app/cogeval/id1366437045). This assessment tool was developed to evaluate cognitive function in patients with MS and was validated against SDMT [3,6]. Demographically adjusted standardized CogEval z-score for Japanese patients with MS was used in this study [7]. The z-score was obtained based on data regarding age and educational background for each patient.

Brain volumetric measurements

Participants were evaluated using a whole-body 1.5 Tesla magnetic resonance imaging system (MAGNETOM Aera, Siemens, Germany). The MR acquisition protocol included (1) a high-resolution sagittal three-dimensional (3D) T1-weighted magnetization-prepared rapid gradient-echo (MPRAGE) sequence (repetition time [TR]: 2730 ms; echo time: 3.3 ms; inversion time [TI]: 1,000 ms; 176 slices; field of view [FoV]: 256 mm; and measured isotropic voxel size: 1×1×1 mm) and (2) a sagittal 3D fluid-attenuated inversion recovery (FLAIR) sequence (TR: 5,000 ms; TE: 335 ms; TI: 1,800 ms; 176 slices; FoV: 256 mm; and measured isotropic voxel size: 1×1×1 mm). The regional and whole-brain volumes were estimated using the automated FreeSurfer stream (version 7.1.0; https://surfer.nmr.mgh.harvard.edu/) [8-11]. The brain volume data extracted from FreeSurfer’s automated segmentation results were normalized to their head size using the estimated total intracranial volume. The unitless derivatives were used in the univariate and multivariable analyses. MS lesion volume load was analyzed with the 3D T1 MPRAGE and 3D FLAIR datasets. The program “icobrain ms” was utilized by uploading the DICOM data to the Icometrix website (http://icometrix.com) [8-10].

Statistical analysis

Continuous variables are described as medians and interquartile ranges (IQRs: 25-75 percentiles). Continuous variables were compared using the Mann-Whitney’s U test. Correlations between the outcome variable (CogEval z-score) and each explanatory variable were evaluated by calculating Pearson’s correlation coefficient (r) and Spearman’s rho (ρ). Multiple linear regression analysis with the CogEval z-score as the dependent (outcome) variable was performed using the characteristics with p<0.10 in the preliminary bivariate correlation analyses as the independent (explanatory) variables. All eligible explanatory variables were entered simultaneously into the regression model. To evaluate the possible bias derived from multicollinearity, the variance inflation factor (VIF) was obtained for each of the used explanatory variables. The VIFs greater than 5 were considered to indicate the presence of multicollinearity that may compromise the estimation of the correlation coefficients. Statistical significance was set at p<0.05. The R statistical software version 4.1.3 (R Foundation for Statistical Computing, Vienna, Austria) was used for all statistical analyses.

Ethics

This study was approved by the Institutional Review Board of the Tohoku Medical and Pharmaceutical University Graduate School of Medicine (approval number: 2017-2-011). All participants provided written informed consent. The study was conducted in accordance with the latest version of the Declaration of Helsinki, as revised in 2013.

## Results

Demographics and CogEval z-scores

Among the 80 patients with MS (19 men and 61 women), 64 (15 men and 49 women) had RRMS and 16 (4 men and 12 women) had SPMS. The median (IQR) age at the CogEval measurement was 43 (37-49) years. The median (IQR) years of disease duration of MS at CogEval was 11 (8-17) years. The median (IQR) scores of the Expanded Disability Status Scale at CogEval was 2.0 (1.0-3.0). As for the disease-modifying drugs used at the CogEval, 32 were with fingolimod, 25 were with dimethyl fumarate, 15 were with natalizumab, two were with interferon-β, one was with ofatumumab, and five were not treated. The CogEval z-scores did not significantly differ between the 19 male and 61 female patients (p=0.4555, Mann-Whitney U test).

**Figure 1:**
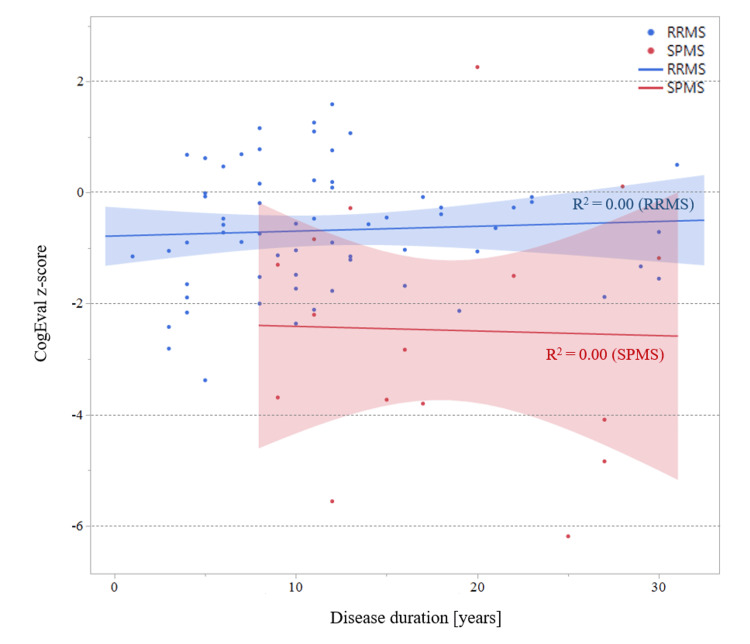
Scatterplots of the disease duration and CogEval z-score in RRMS and in SPMS Scatterplots were created to demonstrate the 64 patients with RRMS (blue plots and line) and 16 patients with SPMS (red plots and line). Colored areas indicate the 95% confidence interval ranges for the approximation line in each disease stage group. In each disease group, disease duration was not associated with the CogEval z-score (R2=0.00 in both the RRMS and SPMS groups). RRMS, relapsing-remitting multiple sclerosis; SPMS, secondary progressive multiple sclerosis

Between the 64 patients with RRMS and 16 with SPMS, the CogEval z-scores were significantly lower in those with SPMS (median [IQR] of −0.675 [−1.51, +0.065] vs −2.52 [−4.02, −0.925]; p=0.0011, Mann-Whitney U test). This finding was confirmed even after adjusting for the age and sex (p<0.0001, analysis of covariance). A scatterplot with the disease duration and CogEval z-score after a stratification by disease stage (RRMS vs SPMS) is presented in Figure 1. In each of the RRMS and SPMS disease stages, disease duration was not associated with the CogEval z-score (R2=0.00 in both groups).

Correlation with CogEval z-score in overall MS

Subsequently, Pearson’s correlation coefficients (r) and Spearman’s rho with the CogEval z-scores were calculated for each of the evaluated explanatory variables (Table 1). The FLAIR-high lesion volume (r= −0.43, p=0.0001), T1-low lesion volume (r= −0.47, p<0.0001), and atrophies in the cerebral cortex (r=0.40, p=0.0002), cerebral white matter (WM) (r=0.48, p<0.0001), corpus callosum (r=0.47, p<0.0001), thalamus (r=0.43, p<0.0001), putamen (r=0.54, p<0.0001), pallidum (r=0.33, p=0.0027), nucleus accumbens (r=0.30, p=0.0072), and cerebellum WM (r=0.33, p=0.0026) indicated a statistically significant correlation (p<0.05) that was evaluated using Pearson’s correlation coefficient. All of these variables achieved statistical significance in Spearman’s rho, except for the atrophies in pallidum (ρ=0.22, p=0.055), nucleus accumbens (ρ=0.21, p=0.067), and cerebellum WM (ρ=0.20, p=0.074).

**Table 1:** Bivariate correlation coefficients between the CogEval z-scores and each evaluated characteristic Pearson’s correlation coefficients (r) and Spearman’s rho (ρ) with the CogEval z-score were calculated from the data of the 80 patients with MS (64 with RRMS and 16 with SPMS). A p-value of 0.05 or less was considered statistically significant. FLAIR, fluid-attenuated inversion recovery; WM, white matter; MS, multiple sclerosis; RRMS, relapsing-remitting multiple sclerosis; SPMS, secondary progressive multiple sclerosis *Volumes adjusted for the estimated total intracranial volume

Characteristics	Pearson’s r [95% CI]	p-Value	Spearman’s ρ	p-Value
Age	–0.079 [–0.29, +0.14]	0.49	+0.017	0.88
Disease duration	–0.13 [–0.34, +0.092]	0.25	–0.043	0.71
FLAIR-high lesion load	–0.43 [–0.60, –0.22]	0.0001	–0.40	0.0004
T1-low lesion load	–0.47 [–0.63, –0.27]	<0.0001	–0.39	0.0006
Whole brain volume	+0.46 [+0.26, +0.63]	<0.0001	+0.35	0.0023
Cerebral cortex*	+0.40 [+0.20, +0.57]	0.0002	+0.24	0.031
Cerebral WM*	+0.48 [+0.29, +0.63]	<0.0001	+0.28	0.013
Corpus callosum*	+0.47 [+0.28, +0.63]	<0.0001	+0.36	0.0011
Thalamus*	+0.43 [+0.23, +0.59]	<0.0001	+0.36	0.0010
Caudate*	+0.22 [–0.002, +0.42]	0.052	+0.16	0.16
Putamen*	+0.54 [+0.36, +0.68]	<0.0001	+0.40	0.0002
Pallidum*	+0.33 [+0.12, +0.51]	0.0027	+0.22	0.055
Hippocampus*	+0.044 [–0.18, +0.26]	0.70	+0.013	0.91
Amygdala*	+0.10 [–0.12, +0.32]	0.36	+0.051	0.65
Nucleus accumbens*	+0.30 [+0.084, +0.49]	0.0072	+0.21	0.067
Cerebellum cortex*	+0.19 [–0.034, +0.39]	0.096	+0.16	0.16
Cerebellum WM*	+0.33 [+0.12, +0.51]	0.0026	+0.20	0.074

Multivariable analyses with the CogEval z-score

To validate the significance of thalamus and putamen volumes for the CogEval z-scores independent of other brain regions, multiple linear regression analyses were performed among the 80 patients using the CogEval z-scores as the outcome variable and the characteristics with p<0.10 in the bivariate correlation analyses (FLAIR-hyperintense lesion load, cerebral cortex, cerebral WM, corpus callosum, cerebellum WM, nucleus accumbens, and either of thalamus or putamen) as the explanatory variables (Table 2). T1-hypointense lesion load was not included because of its strong positive correlation with FLAIR-hyperintense lesion load. Consequently, putamen atrophy was significantly associated with the CogEval z-score independent of other explanatory variables (standardized beta coefficient=0.456, p=0.038). Meanwhile, thalamic atrophy was not significantly associated with the CogEval z-score independent of the three volumes (standardized beta coefficient=0.050, p=0.79).

**Table 2:** Multiple linear regression analysis for the CogEval z-scores To validate the significance of thalamus and putamen volumes for the CogEval z-scores (dependent variable) independent of other brain regions, multiple linear regression analysis was performed by simultaneously using the characteristics with p<0.10 in the bivariate correlation analyses (FLAIR-hyperintense lesion load, cerebral cortex, cerebral WM, corpus callosum, cerebellum WM, nucleus accumbens, and either of thalamus or putamen) as the explanatory variables with the 80 patients. T1-hypointense lesion load was not included because of its strong positive correlation with FLAIR-hyperintense lesion load, resulting in a VIF of greater than 10. Upper half of the table included the putamen volume in the explanatory variables and the lower half included the thalamic volume. A p-value of 0.05 or less was considered statistically significant. VIF, variance inflation factor; FLAIR, fluid-attenuated inversion recovery; WM, white matter *Volumes adjusted for the estimated total intracranial volume

Characteristics	Standardized coefficient (β)	t-score	p-Value	VIF
Regression model including the thalamic volume				
FLAIR-hyperintense lesion	–0.077	–0.47	0.64	2.590
Cerebral cortex*	0.297	2.11	0.038	1.911
Cerebral WM*	0.120	0.59	0.56	4.031
Corpus callosum*	0.209	1.16	0.25	3.167
Cerebellum WM*	0.168	1.18	0.24	1.977
Nucleus accumbens*	–0.233	–1.51	0.14	2.326
Thalamus*	0.050	0.27	0.79	3.383
Regression model including the putamen volume				
FLAIR-hyperintense lesion	–0.003	–0.02	0.99	2.623
Cerebral cortex*	0.194	1.36	0.18	2.112
Cerebral WM*	–0.021	–0.10	0.92	4.459
Corpus callosum*	0.223	1.28	0.21	3.140
Cerebellum WM*	0.088	0.66	0.51	1.841
Nucleus accumbens*	–0.302	–1.99	0.051	2.395
Putamen*	0.456	2.11	0.038	4.833

Subgroup analysis in RRMS

To evaluate the significance of atrophy in the putamen in MS progression, Pearson’s correlation coefficients (r) and Spearman’s rho (ρ) between CogEval z-scores and each evaluated characteristic was calculated in the 64 patients with RRMS (Table 3). None of the evaluated explanatory variables demonstrated significant correlations with the CogEval z-scores, suggesting that an impaired cognitive processing speed with brain regional atrophies are not remarkable in the early stages of MS.

**Table 3:** Bivariate correlation coefficients with the CogEval z-score in RRMS Pearson’s correlation coefficients (r) and Spearman’s rho (ρ) between the CogEval z-score and each evaluated explanatory variable were calculated among the 64 patients with RRMS. A p-value of 0.05 or less was considered statistically significant. FLAIR, fluid-attenuated inversion recovery; WM, white matter; RRMS, relapsing-remitting multiple sclerosis *Volumes adjusted for the estimated total intracranial volume

Characteristics	Pearson’s r [95% CI]	p-Value	Spearman’s ρ	p-Value
Age	+0.11 [–0.14, +0.34]	0.41	+0.15	0.25
Disease duration	+0.060 [–0.19, +0.30]	0.64	+0.12	0.36
FLAIR-high lesion load	–0.21 [–0.44, +0.052]	0.12	–0.20	0.13
T1-low lesion load	–0.23 [–0.46, +0.035]	0.089	–0.20	0.14
Whole brain volume	+0.12 [–0.14, +0.37]	0.38	+0.102	0.45
Cerebral cortex*	+0.11 [–0.14, +0.34]	0.39	+0.095	0.45
Cerebral WM*	+0.066 [–0.18, +0.31]	0.60	+0.031	0.81
Corpus callosum*	–0.18 [–0.41, +0.071]	0.16	+0.10	0.41
Thalamus*	+0.19 [–0.061, +0.41]	0.14	+0.20	0.11
Caudate*	–0.010 [–0.26, +0.24]	0.94	+0.021	0.87
Putamen*	+0.23 [–0.014, +0.45]	0.064	+0.19	0.13
Pallidum*	–0.002 [–0.25, +0.24]	0.99	–0.009	0.95
Hippocampus*	–0.063 [–0.30, +0.19]	0.62	–0.067	0.60
Amygdala*	–0.026 [–0.27, +0.22]	0.84	–0.025	0.84
Nucleus accumbens*	+0.016 [–0.23, +0.26]	0.90	+0.000	>0.99
Cerebellum cortex*	+0.11 [–0.14, +0.34]	0.40	+0.059	0.64
Cerebellum WM*	+0.020 [–0.23, +0.26]	0.88	–0.028	0.83

Subgroup analysis in SPMS

Finally, univariate correlation analyses were further performed within the 16 patients with SPMS as a sensitivity analysis (Table 4). Atrophies in the cerebral WM (r=0.67, p=0.005), corpus callosum (r=0.72, p=0.002), thalamus (r=0.53, p=0.034), putamen (r=0.66, p=0.0054), and pallidum (r=0.56, p=0.023) demonstrated statistically significant Pearson’s correlation coefficients with impaired cognitive processing speed even among the patients with SPMS. These brain regions demonstrated significant correlations with the CogEval z-scores even when the correlations were assessed using Spearman’s rho, only except for atrophy in the thalamus (ρ=0.41, p=0.12).

**Table 4:** Bivariate correlation coefficients with CogEval z-score in SPMS Pearson’s correlation coefficients between the CogEval z-score and each evaluated explanatory variable were calculated among the 16 patients with SPMS. Atrophies in the thalamus, putamen, and pallidum were significantly correlated with the impaired cognitive processing speed in the 16 patients with SPMS. A p-value of 0.05 or less was considered statistically significant. FLAIR, fluid-attenuated inversion recovery; WM, white matter; SPMS, secondary progressive multiple sclerosis *Volumes adjusted for the estimated total intracranial volume

Characteristics	Pearson’s r [95% CI]	p-Value	Spearman’s ρ	p-Value
Age	–0.17 [–0.61, +0.36]	0.54	–0.19	0.49
Disease duration	–0.027 [–0.52, +0.48]	0.92	–0.040	0.88
FLAIR-high lesion load	–0.41 [–0.76, +0.13]	0.13	–0.45	0.090
T1-low lesion load	–0.47 [–0.79, +0.060]	0.080	–0.50	0.058
Whole brain volume	+0.65 [+0.21, +0.87]	0.0084	+0.57	0.028
Cerebral cortex*	+0.41 [–0.11, +0.75]	0.12	+0.41	0.11
Cerebral WM*	+0.67 [+0.26, +0.87]	0.0047	+0.64	0.0082
Corpus callosum*	+0.72 [+0.36, +0.90]	0.0015	+0.72	0.0015
Thalamus*	+0.53 [+0.051, +0.81]	0.034	+0.41	0.12
Caudate*	+0.24 [–0.29, +0.66]	0.37	+0.23	0.39
Putamen*	+0.66 [+0.24, +0.87]	0.0054	+0.62	0.011
Pallidum*	+0.56 [+0.095, +0.83]	0.023	+0.59	0.017
Hippocampus*	–0.21 [–0.64, +0.32]	0.44	–0.27	0.31
Amygdala*	+0.031 [–0.47, +0.52]	0.91	–0.041	0.88
Nucleus accumbens*	+0.29 [–0.24, +0.69]	0.27	+0.30	0.26
Cerebellum cortex*	–0.19 [–0.63, +0.33]	0.47	–0.16	0.56
Cerebellum WM*	+0.42 [–0.097, +0.76]	0.11	+0.32	0.22

## Discussion

In this study, a significant correlation between putamen atrophy and impaired cognitive processing speed was confirmed in the patients with MS. This association was not apparent in the earlier disease stages with relapsing-remitting disease course but was remarkable in the later disease stages after a transition to SPMS. To the best of our knowledge, this study is the first to demonstrate the potential role of putamen atrophy in the progression of higher cognitive dysfunction in patients with MS. In addition to the putamen volume, the corpus callosum volume may also influence the cognitive processing speed in the later stages of MS, although the correlation in the multiple linear regression analyses did not reach statistical significance. Certainly, atrophies in the cerebral cortex and cerebral WM were among the changes accompanying the impaired higher brain function in later MS, but atrophies in more specific brain components, such as the putamen, are also present and may be more primarily associated with the impaired cognitive processing in MS.

CI is generally more prominent in patients with progressive forms of MS than in those with the relapsing-remitting form, possibly owing to the extensive neurodegeneration [2]. The performance of SDMT is reported to be the earliest to decline among several cognitive tests for MS [12-15] and has been demonstrated to gradually decline over time [15]. Thalamic atrophy has been reported to correlate with SDMT performance [16-18], which could be among the mechanisms underlying the early decline of cognitive performance in MS [15,19]. However, poor performance on the SDMT is associated with multiple brain pathologies, such as diffuse WM damage or reduced volumes in the deep gray matter, cerebellum, and several cerebral cortical regions [1,15,18,20-24]. Consequently, SDMT is influenced by multiple factors other than IPS, including attention, working memory, and mental flexibility [25,26]. The SDMT can be further influenced by learning ability executive functions, as has been documented in SPMS [1]. Poor performance on the SDMT can be accelerated in the late phase of MS [15].

Although most of the previous studies have suggested that the thalamus is the most important neural substrate for SDMT performance, our results indicated the potential importance of the putamen. We considered that this discrepancy might be caused by several methodological factors. For example, our cohort included both patients with RRMS and SPMS, suggesting that a long disease duration in some patients of this study could have produced the finding. The correlations between CogEval z-score and thalamic volume were not significant in 27 patients with RRMS with a disease duration of <10 years (Spearman's rho=0.24; p=0.23) or in nine patients with RRMS with a disease duration of <5 years (Spearman's rho=0.067; p=0.86). Similarly, the correlations between CogEval z-score and putamen volume were not significant in 27 patients with RRMS with a disease duration of <10 years (Spearman's rho=0.25; p=0.20) or in nine patients with RRMS with a disease duration of <5 years (Spearman's rho=0.13; p=0.73). These findings indicate that the association between thalamic or putamen atrophy and poor PST performance in MS becomes clear in the context of long-term observation periods. To identify the specific brain regions related to poor PST performance in early disease stages, a larger sample size will be needed.

Our results indicated that the progressive putamen atrophy may affect the gradual decline of SDMT performance during the long-term observational periods. Although the exact mechanisms remain uncertain, several theories might be considered. For example, in a study that examined MS-specific atrophy (i.e., atrophy in excess of normal aging), the contribution of normal aging has been demonstrated for thalamic atrophy, but it was not demonstrated for putamen atrophy [27]. Therefore, contribution of MS-specific thalamic atrophy on the SDMT performance might decline as the patients’ age increases, whereas that of the putamen atrophy does not. Another recent study demonstrated different SDMT performances between early, intermediate, and late stages of brain volume loss [15]. The study also revealed that the volume of the putamen was significantly different in the three stages, whereas that of the thalamus was significantly different only between the early and intermediate stages. These results suggest that the correlation between putamen volume and SDMT score might be more remarkable than that of thalamic volume in the later stage of MS. Furthermore, several previous studies have reported the potential role of putamen damage in the disturbed IPS in patients with MS [18,28-30]. Putamen is involved in voluntary fixational control and saccades and contributes to planning tasks with a visual searching component. A previous study suggested the role of the putamen in IPS deficits, emphasizing the role of visual movements in cognitive tasks influenced by visual searching such as SDMT [18,28]. Similar to SDMT, the PASAT is also a useful cognitive tool with high sensitivity to sustained attention and IPS alterations, although the PASAT involves several different cognitive functions from SDMT, such as auditory perception and processing, speech production, and mathematical abilities. To date, several studies have reported that the PASAT score is associated with volumes of several subcortical structures, including the putamen [28,31]. The supposed scenario for the pathogenesis of MS-related CI would not be simple, probably based on the focal and diffuse involvement of both the white matter and gray matter, together with pathological changes in specific cortical and subcortical central nervous system structures [1]. Trying to link a specific cognitive domain to a single specific brain area might be an inappropriate approach, as a specific cognitive domain is supported by multiple brain structures, and individual neuronal networks support multiple cognitive domains [1]. Therefore, we emphasize the importance of the putamen, in addition to the thalamus, as one of the substrates and imaging biomarkers underlying the SDMT performance in a long-term observation of patients with MS.

This study had several limitations. First, this study did not evaluate the physical and mental conditions that may affect the cognitive profiles in patients with MS, such as depression, anxiety, fatigue, and sleep disturbances [32]. For example, increased depressive symptoms in patients with MS over time have been associated with decreased processing speed [33,34]. Additionally, a strong negative correlation has been identified between fatigue and processing speed in MS [35]. To date, several studies have suggested that MS-related fatigue is associated with the caudate, putamen, pallidum, and pons [36], or striato-thalamo-cortical network [37-39]. Moreover, neuroimaging studies in patients with MS with fatigue, depression, and pain have indicated gray matter atrophy and decreased functional connectivity in the prefrontal cortex, basal ganglia, and limbic system that are core areas of the mesocorticolimbic system, a key structure in valence and reward processing [40]. Thus, the atrophy of the putamen may have affected the results of the SDMT via the complication of depression or fatigue. Finally, the generalizability of the findings to other races remains unknown. For example, a recent study revealed that the PST scores in Japanese volunteers differed from those of the age-restricted and propensity score-matched U.S. cohort. Japanese people use Kanji in writing the language, which is a logographic system of Chinese characters. Visual memory is supposedly instrumental in literacy acquisition of logographic Kanji from childhood [41], and this early life visual memory exercise may contribute to a facilitated processing speed in the PST among Japanese [7].

## Conclusions

A progression of impaired cognitive processing speed was confirmed in the later disease stages of MS. Among the evaluated brain regions, putamen atrophy was suggested as a primary finding underlying the impaired cognitive processing speed in the disease. This finding was more remarkable in later disease stages after a transition to SPMS than in earlier disease stages. Further studies are needed to elucidate the role of atrophy in putamen in the mechanisms of MS.
